# Supplementary figures and images for: S100 Calcium-Binding Protein P Secreted from Megakaryocytes Promotes Osteoclast Maturation
Source: Int J Mol Sci. 2021 Jun 7;22(11):6129. doi: 10.3390/ijms22116129 (PMC8201154; doi:10.3390/ijms22116129)

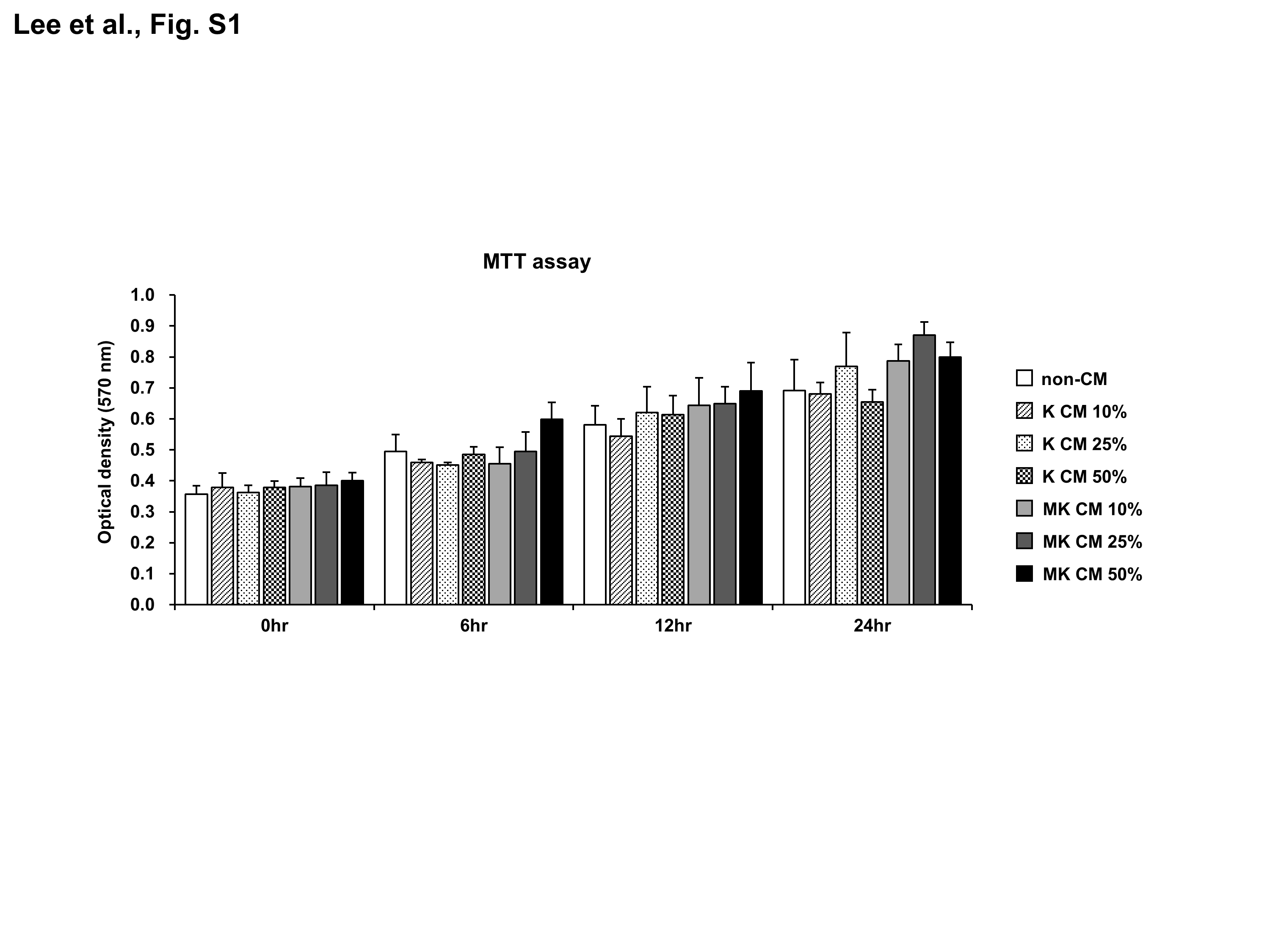

Supplement: Supplementary file 1 [file ijms-22-06129-s001.zip › Lee et al -Supplementary Fig S1.tif]

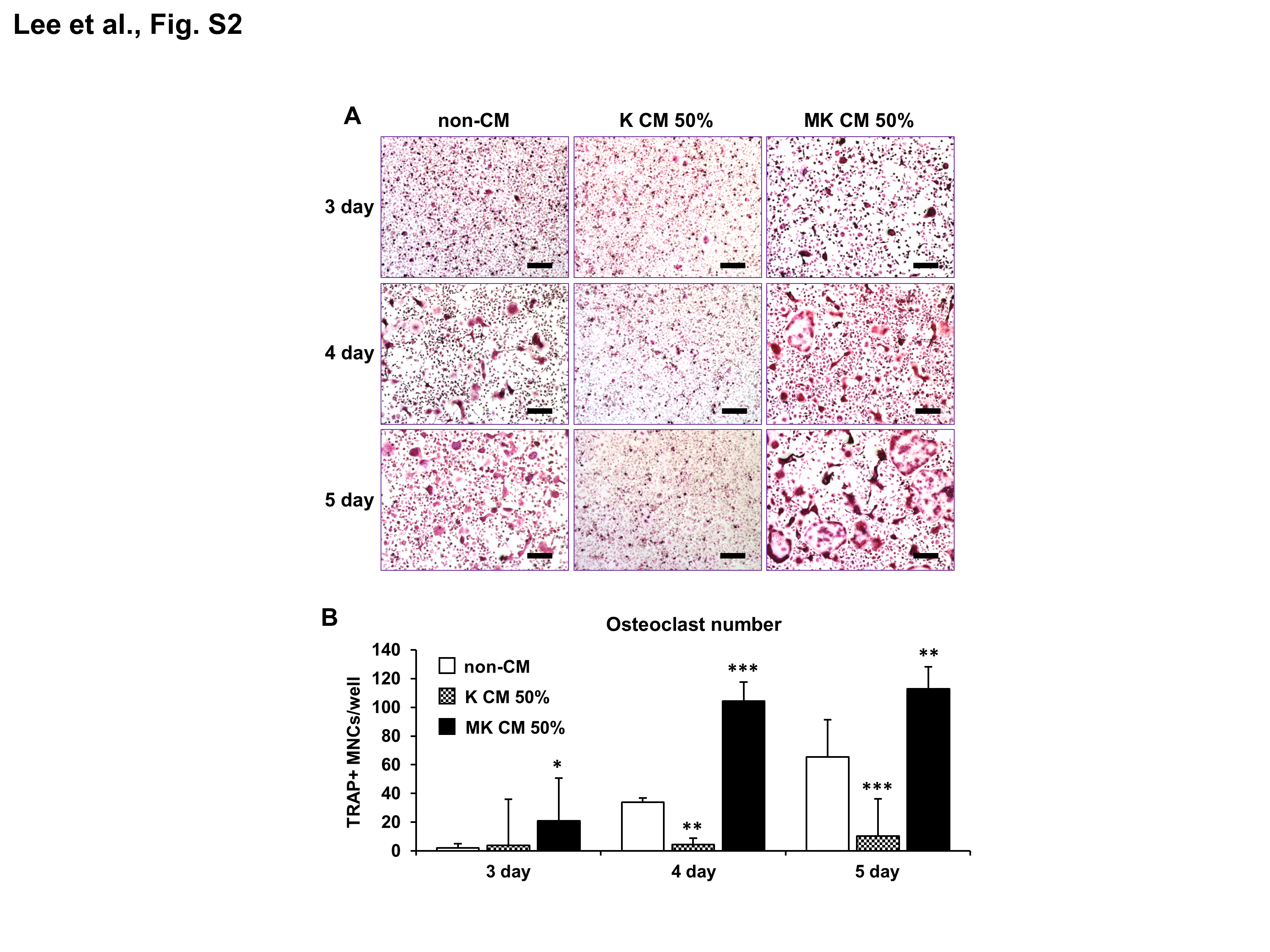

Supplement: Supplementary file 1 [file ijms-22-06129-s001.zip › Lee et al -Supplementary Fig S2.tif]

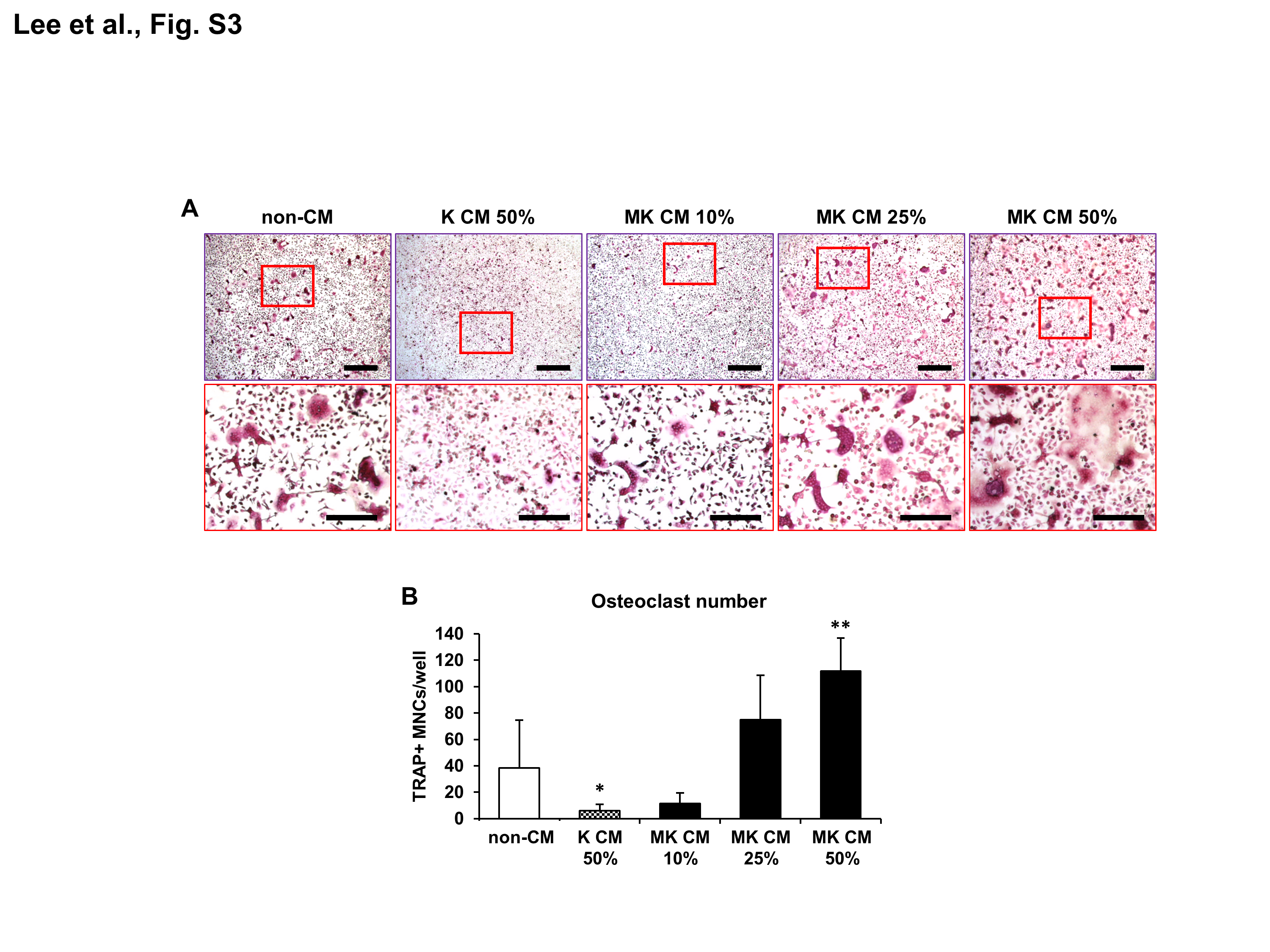

Supplement: Supplementary file 1 [file ijms-22-06129-s001.zip › Lee et al -Supplementary Fig S3.tif]
